# Cytotoxic potential of selected medicinal plants in northeast Brazil

**DOI:** 10.1186/s12906-016-1166-1

**Published:** 2016-07-08

**Authors:** Thiago B. C. da Silva, Cinara O. D’Sousa Costa, Alexandre F. C. Galvão, Larissa M. Bomfim, Ana Carolina B. da C. Rodrigues, Mauricio C. S. Mota, Alex A. Dantas, Tiago R. dos Santos, Milena B. P. Soares, Daniel P. Bezerra

**Affiliations:** Undade de Ensino de Viçosa, Campus Arapiraca, Universidade Federal de Alagoas, Viçosa, Alagoas Brazil; Instituto Gonçalo Moniz, Fundação Oswaldo Cruz (IGM/FIOCRUZ-BA), Rua Waldemar Falcão, 121, Candeal, 40296-710 Salvador, Bahia Brazil; Centro de Biotecnologia e Terapia Celular, Hospital São Rafael, Salvador, Bahia Brazil

**Keywords:** Cytotoxicity, Northeastern Brazil, Medicinal plants, Natural products

## Abstract

**Background:**

Great biodiversity is a highlight of Brazilian flora. In contrast, the therapeutic potentialities of most species used in folk medicine remain unknown. Several of these species are commonly used to treat cancer. In this study, we investigated the cytotoxic activity of 18 plants from 16 families that are found in the northeast region of Brazil.

**Methods:**

The following species were studied: *Byrsonima sericea* DC. (Malpighiaceae), *Cupania impressinervia* Acev. Rodr. var. (revoluta) Radlk (Sapindaceae), *Duranta repens* Linn. (Verbenaceae), *Helicostylis tomentosa* (Poepp. & Endl) Rusby (Moraceae), *Himatanthus bracteatus* (A.DC.) Woodson (Apocynaceae), *Ipomoea purga* (Wender.) Hayne (Convolvulaceae), *Ixora coccinea* Linn. (Rubiaceae), *Mabea piriri* Aubl. (Euphorbiaceae), *Miconia minutiflora* (Melastomataceae), *Momordica charantia* L. (Cucurbitaceae), *Ocotea glomerata* (Nees) Mez (Lauraceae), *Ocotea longifolia* Kunth (*Oreodaphne opifera* Mart. Nees) (Lauraceae), *Pavonia fruticosa* (Mill.) Fawc. & Rendle (Malvaceae), *Psychotria capitata* Ruiz & Pav. (Rubiaceae), *Schefflera morototoni* (Aubl.) Maguire, Steyerm. & Frodin (Araliaceae), *Solanum paludosum* Moric. (Solanaceae), *Xylopia frutescens* Aubl. (Annonaceae) and *Zanthoxylum rhoifolium* Lam. (Rutaceae). Their dried leaves, stems, flowers or fruits were submitted to different solvent extractions, resulting in 55 extracts. After incubating for 72 h, the cytotoxicity of each extract was tested against tumor cell lines using the alamar blue assay.

**Results:**

The *B. sericea, D. repens, H. bracteatus, I. purga, I. coccinea, M. piriri, O. longifolia* and *P. capitata* extracts demonstrated the most potent cytotoxic activity. The chloroform soluble fractions of *D. repens* flowers and the hexane extract of *I. coccinea* flowers led to the isolation of quercetin and a mixture of α- and β-amyrin, respectively, and quercetin showed moderate cytotoxic activity.

**Conclusion:**

The *B. sericea, D. repens, H. bracteatus, I. purga, I. coccinea, M. piriri, O. longifolia* and *P. capitata* plants were identified as having potent cytotoxic effects. Further investigations are required to determine the mechanisms of cytotoxicity exhibited and their in vivo activities. This work reinforces the need to understand the therapeutics potentialities of Brazilian medicinal plants.

## Background

In Brazil, the estimated number of described plant species (terrestrial and algae) ranges from 50,542 to 60,042 [1]; they are found in the following six different biomes: Amazônia (the Amazon rainforest in northern and central-western Brazil), Cerrado (the central Brazilian savanna), Mata Atlântica (the Atlantic rainforest ranging from sea level to the eastern highlands of Brazil), Caatinga (a xerophilous thorny forest found in northeastern Brazil), Pampa (the grasslands in southern Brazil) and Pantanal (periodically flooded grasslands by the Paraná and Paraguay rivers in central-western Brazil). Thus, Brazil is one of the most biodiverse nations in the world [1–3].

The cytotoxic potential of some Brazilian medicinal plants has been previously investigated [4–7]; however, due to the country’s great biodiversity, the therapeutic potentialities of most species remain unknown. In this work, the cytotoxic activities of 18 plants belonging to 16 families found in northeast Brazil were assessed against tumor cell lines. The following species were studied: *Byrsonima sericea* DC. (Malpighiaceae), *Cupania impressinervia* Acev. Rodr. var. (revoluta) Radlk (Sapindaceae), *Duranta repens* Linn. (Verbenaceae), *Helicostylis tomentosa* (Poepp. & Endl) Rusby (Moraceae), *Himatanthus bracteatus* (A.DC.) Woodson (Apocynaceae), *Ipomoea purga* (Wender.) Hayne (Convolvulaceae), *Ixora coccinea* Linn. (Rubiaceae), *Mabea piriri* Aubl. (Euphorbiaceae), *Miconia minutiflora* (Melastomataceae), *Momordica charantia* L. (Cucurbitaceae), *Ocotea glomerata* (Nees) Mez (Lauraceae), *Ocotea longifolia* Kunth (*Oreodaphne opifera* Mart. Nees) (Lauraceae), *Pavonia fruticosa* (Mill.) Fawc. & Rendle (Malvaceae), *Psychotria capitata* Ruiz & Pav. (Rubiaceae), *Schefflera morototoni* (Aubl.) Maguire, Steyerm. & Frodin (Araliaceae), *Solanum paludosum* Moric. (Solanaceae), *Xylopia frutescens* Aubl. (Annonaceae) and *Zanthoxylum rhoifolium* Lam. (Rutaceae). The plant species, family, vernacular name and traditional use in Brazil are shown in Table 1.

**Table 1:** List of plants and their vernacular name and folk use in Brazil

Plant species	Family	Vernacular name	Folk use in Brazil
*Byrsonima sericea* DC.	Malpighiaceae	Murici-da-mata	A decoction of the stem-bark is used to treat fevers, diarrhea, syphilis and kidney disease [26]
*Cupania impressinervia* Acev. Rodr. var. (revoluta) Radlk	Sapindaceae	Caboatã-de-rego	-
*Duranta repens* Linn.	Verbenaceae	Pingo-de-ouro	Ornamental [27]
*Helicostylis tomentosa* (Poepp. & Endl) Rusby	Moraceae	Amoreira-preta	Its fruit is used as a nutritional source [28]
*Himatanthus bracteatus* (A.DC.) Woodson	Apocynaceae	Janaguba	The latex is topically applied to treat external ulcers and tumors. It is dropped in a liter of water against inflammation and cancer [26]
*Ipomoea purga* (Wender.) Hayne	Convolvulaceae	Jalapa, batata-de-purga	It is used as an analgesic [29]
*Ixora coccinea* Linn.	Rubiaceae	Ixora	Ornamental [30]
*Mabea piriri* Aubl.	Euphorbiaceae	Canudo de cachimbo	-
*Miconia minutiflora*	Melastomataceae	Mundururu	Its fruit is used as a nutritional source [28]
*Momordica charantia* L.	Cucurbitaceae	Melão-de-sabiá, melão-de-são-caetano, galinha-de-melão	A decoction of a handful in a liter of water is used as a tea to treat diabetes, parasite infections, rheumatism and diarrhea [26]
*Ocotea glomerata* (Nees) Mez	Lauraceae	Caneleira, louro-branco, louro-abacate, louro-bravo	The leaves are used to treat hydropsy, digestive problems and high blood pressure [31, 32]
*Ocotea longifolia* Kunth (*Oreodaphne opifera* Mart. Nees)	Lauraceae	Canela de cheiro	Fruit oil is used to treat arthralgia and rheumatic diseases [33]
*Pavonia fruticosa* (Mill.) Fawc. & Rendle	Malvaceae	-	-
*Psychotria capitata* Ruiz & Pav.	Rubiaceae	-	-
*Schefflera morototoni* (Aubl.) Maguire, Steyerm. & Frodin	Araliaceae	Matatauba, Marupá	Its fruit is used as a nutritional source and to make local crafts [28]
*Solanum paludosum* Moric.	Solanaceae	Jurubeba-brava, jurubeba-roxa	An infusion of the roots is used to treat hepatic diseases. The fruit is used as a poison [26]
*Xylopia frutescens* Aubl.	Annonaceae	Embira, semente-de-embira	Its seeds and fruits are used as a digestive aid (a decoction of a teaspoon in a cup of water). It is used as a tea after meals [26]
*Zanthoxylum rhoifolium* Lam.	Rutaceae	Mamica de cadela, mamica de porca	The roots are used as a febrifuge, digestant and tonic. The stem bark is used to treat flatulence, colic, dyspepsia, ear aches, toothaches and snake bites [34]

## Methods

### Plant material

Leaves, stems, fruits and/or flowers of *B. sericea* (voucher number 45628)*, C. impressinervia* (voucher number 42953)*, D. repens* (voucher number 25870)*, H. tomentosa* (voucher number 20586)*, H. bracteatus* (voucher number 50733)*, I. purga* (voucher number 33255) and *I. coccinea* (voucher number 25861) were collected in February 2014 in the Municipality of Viçosa, Alagoas, Brazil. Leaves and/or stems of *M. piriri* (voucher number 45717)*, M. minutiflora* (voucher number 49653)*, M. charantia* (voucher number 34393)*, O. glomerata* (voucher number 50577)*, O. longifolia* (voucher number 50577)*, P. fruticosa* (voucher number 45659) and *P. capitata* (voucher number 45620) were collected in March 2014 in the municipality of Rio Largo, Alagoas, Brazil. Leaves, stems and/or fruits of *S. morototoni* (voucher number 45659)*, S. paludosum* (voucher number 37400)*, X. frutescens* (voucher number 45677) and *Z. rhoifolium* (voucher number 49656) were collected in March 2014 in the Municipality of Murici, Alagoas, Brazil. All plants were collected in the Mata Atlântica bioma. The species were identified by Rosângela Pereira e Lyra Lemos, a plant taxonomist from the Instituto do Meio Ambiente de Alagoas IMA-AL. Voucher botanical specimens are stored at the Herbarium of the Instituto do Meio Ambiente de Alagoas IMA-AL. This work was performed according to the special authorization for access to genetic resources in Brazil # 010344/2014-4, issued by CNPq/MCTI.

### Plant extractions

The dried leaves, stems, flowers or fruits were triturated and extracted at room temperature with ethanol or hexane. The solvent was removed to produce the correspondent crude extracts. The extraction steps for each sample were repeated three times with one-week intervals. Part of leaves, fruits or flowers ethanol extract was suspended in methanol:water (9:1) solution and extracted successively with ethyl acetate, chloroform, hexane or hexane:ethyl acetate (1:1) to produce the corresponding partitioned extracts.

### Chemical fractionation

#### General procedures

Purification processes were performed through column chromatography (CC) using silica gel 60 (0.063-0.200 mm) as the stationary phase. Organic solvents or mixtures of increasing polarity were used as mobile phases. Silica gel 60 GF (Merck, Darmstadt, Germany) was used to perform analytical (0.25 mm) or preparative (0.75 mm) thin layer chromatographic (TLC) processes.

The ^1^H (400 MHz) and ^13^C (100 MHz) nuclear magnetic resonance (NMR) spectra were obtained on a Bruker Avance DRX-400 spectrometer (Bruker, Rheinstetten, Germany), operating at 300 K. The chemical shifts (δ) were expressed in units of ppm, using tetramethylsilane (TMS) as a reference (δ_H_ = δ_c_ = 0), and the coupling constants (*J*) were expressed in Hz. Chloroform-d was used as the solvent for the samples.

### Isolation of compounds

The chloroform extract of *D. repens* flowers (7 g) was submitted to silica gel CC eluted with hexane and ethyl acetate (either pure or a mixture of increasing polarity), providing 50 fractions (5 mL each) that were grouped with hexane and ethyl acetate. After solvent evaporation, fractions 30–50 were purified with Sephadex-L water:methanol, yielding the compound quercetin (**56**, 11.0 mg). The compound structure was identified through a series of spectrometric data, such as NMR, as well as by comparison with data reported in the literature.

A hexane extract of *I. coccinea* flowers (26 g) was submitted to silica gel CC eluted with hexane, ethyl acetate and methanol (either pure or a mixture of increasing polarity), providing 100 fractions (5 mL each) that were grouped with hexane, ethyl acetate and methanol. After solvent evaporation from fractions 30 to 45 (eluted with hexane/ethyl acetate 8:2), a white solid (in the form of flakes) was obtained (**57**, 14.0 mg). This solid was identified as a mixture of α- and β-amyrin. The compounds structures were identified through a series of spectrometric data, such as NMR, as well as by comparison with data reported in the literature.

### Cells

Tumor cells lines B16-F10 (mouse melanoma), HepG2 (human hepatocellular carcinoma), K562 (human chronic myelocytic leukemia) and HL-60 (human promyelocytic leukemia) were donated by the Hospital A.C. Camargo, São Paulo, SP, Brazil. This panel of cell lines include different types of cell histology with different sensibility. Cells were maintained in Roswell Park Memorial Institute-1640 (RPMI-1640, Gibco-BRL, Gaithersburg, USA) medium supplemented with 10 % fetal bovine serum (Cultilab, Campinas, Brazil), 2 mM L-glutamine (Vetec Química Fina, Duque de Caxias, Brazil) and 50 μg/mL gentamycin (Novafarma, Anápolis, Brazil). Adherent cells were harvested by treatment with 0.25 % trypsin EDTA solution (Gibco-BRL, Gaithersburg, USA). All cell lines were cultured in cell culture flasks at 37 °C in 5 % CO_2_ and sub-cultured every 3–4 days to maintain exponential growth. All experiments were conducted with cells in the exponential growth phase. All cell lines were tested for mycoplasma with a Mycoplasma Stain Kit (Sigma-Aldrich, St Louis, MO, USA) and found to be free from contamination.

Heparinized blood (from healthy, 20- to 35-year-old, non-smoking donors who had not taken any drug for at least 15 days prior to the sampling) was collected, and peripheral blood mononuclear cells (PBMC) were isolated using a standard protocol, with Ficoll (Ficoll-Paque Plus, GE Healthcare Bio-Sciences AB, Sweden) density gradient centrifugation. PBMC were washed and resuspended at a concentration of 0.3 × 10^6^ cells/mL in RPMI 1640 medium supplemented with 20 % fetal bovine serum, 2 mM L-glutamine, and 50 μg/mL gentamycin at 37 °C with 5 % CO_2_. In addition, concanavalin A (ConA, Sigma Chemical Co. St Louis, MO, USA) was used as a mitogen to trigger cell division in T-lymphocytes. ConA (10 μg/mL) was added at the beginning of the culture, and after 24 h, the cells were treated with the test drugs.

For all experiments, cell viability was examined using Trypan blue exclusion assays. Over 90 % of the cells were viable at the beginning of the culture.

### Cytotoxicity assay

Cell viability was quantified using alamar blue assay, as previously described [8]. For all experiments, cells were seeded in 96-well plates (0.7 × 10^5^ cells/mL for adherent cells or 0.3 × 10^6^ cells/mL for suspended cells in 100 μL of medium). After 24 h (or immediately for the suspended cells), the samples, which were dissolved in dimethyl sulfoxide (DMSO, Sigma-Aldrich, St Louis, MO, USA) at a final concentration of 50 μg/mL, were added to each well and incubated for 72 h. Doxorubicin (purity >95 %, Laboratórios IMA S.A.I.C., Buenos Aires, Argentina) was used as the positive control. The negative control received the vehicle used to dilute the tested samples (0.5 % DMSO). Four (for cell lines) or 24 h (for PBMC) before the end of the incubation, 20 μL of stock solution (0.312 mg/mL) of the alamar blue (resazurin, Sigma-Aldrich, St Louis, MO, USA) were added to each well. The absorbance was measured using a SpectraMax 190 microplate reader (Molecular Devices, Sunnyvale, USA), and the drug effect was quantified as the percentage of control absorbance at 570 and 600 nm. The extracts that caused more than 75 % cell growth inhibition were tested again at concentrations varying from 0.39 to 50 μg/mL to determine the 50 % inhibitory concentration (IC_50_). Isolated compounds were tested at concentrations varying from 0.19 to 25 μg/mL.

### Statistical analysis

Data are presented as the mean ± S.E.M. The IC_50_ values were obtained through nonlinear regression using the GraphPad program (Intuitive Software for Science, San Diego, USA).

## Results and discussion

**Fig 1:**
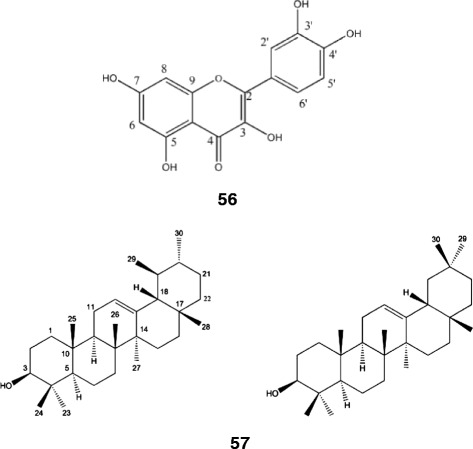
The chemical structures of quercetin (**56**) isolated from the chloroform soluble fractions of *Duranta repens* flowers and α- and β-amyrin (**57**) isolated from the hexane extract of *Ixora coccinea* flowers

Fifty-five extracts were obtained from the plants studied, and their cytotoxicities were tested using the alamar blue assay after 72 h of incubation. The cytotoxicity of each extract was initially tested against two tumor cell lines (HepG2 and HL-60) at a final concentration of 50 μg/mL. The plant species and plant parts/solvent used for extract preparation are shown in Table 2. Concentration-response curves were generated and IC_50_ values were calculated against four tumor cell lines (B16-F10, HepG2, K562 and HL-60) and against one non-tumor cells (PBMC) for the extracts that caused more than 75 % cell growth inhibition (Table 3). In addition, the chloroform soluble fractions of *D. repens* flowers and the hexane extract *I. coccinea* flowers isolated quercetin and the mixture of α- and β-amyrin (Fig. 1), respectively, which were also tested for their cytotoxic activity (Table 3).

**Table 2:** Growth inhibitory effects of plant extracts/fractions against tumor cell lines

No.	Plant species	Parts of the plant^a^ (Extractions^b^)	Cell^c^ growth inhibition percentage (GI %)^d^	
			HepG2	HL60
01	*Byrsonima sericea* DC.	F (e)	25.03 ± 5.24	25.27 ± 8.79
02		S (e)	8.02 ± 6.85	27.32 ± 0.06
03		L (e)	21.84 ± 2.64	64.71 ± 5.28
04		L (m/e)	91.16 ± 0.64	102.7 ± 1.12
05		L (ea/e)	92.14 ± 0.64	103.3 ± 7.26
06		L (c/e)	39.96 ± 9.49	70.12 ± 11.24
07		L (h/e)	91.62 ± 0.39	109.0 ± 2.52
08	*Cupania impressinervia* Acev. Rodr. var. (revoluta) Radlk	L (e)	23.10 ± 5.59	55.19 ± 5.50
09	*Duranta repens* Linn.	FL (m/e)	14.09 ± 7.14	33.3 ± 0.92
10		FL (ea/e)	20.57 ± 7.67	28.36 ± 0.46
11		FL (c/e)	79.94 ± 2.97	98.67 ± 6.35
12	*Helicostylis tomentosa* (Poepp. & Endl) Rusby	S (e)	14.55 ± 3.30	53.67 ± 0,58
13		L (e)	7.96 ± 3.56	38.39 ± 7.33
14	*Himatanthus bracteatus* (A.DC.) Woodson	S (e)	20.55 ± 1.61	29.38 ± 1.63
15		L (e)	−9.90 ± 6.56	−16.91 ± 4.61
16		L (h)	14.78 ± 5.22	70.63 ± 2.62
17		L (m/e)	−0.77 ± 2.61	28.55 ± 3.25
18		L (ea/e)	5.70 ± 2.61	56.16 ± 9.49
19		L (c/e)	92.30 ± 1.50	93.63 ± 0.30
20	*Ipomoea purga* (Wender.) Hayne	S (e)	98.39 ± 1.81	95.58 ± 1.33
21		L (e)	93.86 ± 2.70	106.4 ± 3.62
22	*Ixora coccinea* Linn.	F (e)	−2.97 ± 9.22	1.27 ± 3.86
23		FL (h)	21.47 ± 5.44	−25.64 ± 9.33
24		L (m/e)	9.25 ± 4.73	1.38 ± 1.35
25		L (ea/e)	7.49 ± 5.16	32.25 ± 9.39
26		L (h/e)	22.55 ± 3.23	58.78 ± 1.34
27		F (ea/e)	−5.53 ± 7.07	18.83 ± 5.74
28		F (c/e)	94.93 ± 0.74	92.97 ± 2.39
29	*Mabea piriri* Aubl.	S (e)	81.41 ± 8.27	92.35 ± 7.66
30		L (e)	12.48 ± 5.12	69.56 ± 0.17
31	*Miconia minutiflora*	S (e)	25.64 ± 6.34	38.17 ± 1.16
32		L (e)	38.62 ± 2.72	66.54 ± 0.85
33	*Momordica charantia* L.	L (e)	−17.51 ± 2.97	9.30 ± 5.64
34	*Ocotea glomerata* (Nees) Mez	S (e)	49.49 ± 9.87	32.70 ± 9.32
35		L (c/e)	25.48 ± 7.87	52.0 ± 3.12
36	*Ocotea longifolia* Kunth (*Oreodaphne opifera* Mart. Nees)	S (e)	−7.43 ± 5.88	2.49 ± 0.33
37		L (e)	93.88 ± 2.12	94.70 ± 1.06
38	*Pavonia fruticosa* (Mill.) Fawc. & Rendle	S (e)	44.81 ± 1.66	41.65 ± 6.51
39		L (e)	33.34 ± 4.48	55.82 ± 4.66
40	*Psychotria capitata* Ruiz & Pav.	S (e)	15.82 ± 3.12	64.16 ± 0.32
41		L (e)	28.13 ± 8.45	22.00 ± 5.92
42		L (m/e)	19.16 ± 8.90	51.72 ± 4.86
43		L (ea/e)	95.14 ± 1.83	86.55 ± 4.20
44		L (c/e)	96.92 ± 1.68	92.87 ± 0.06
45		L (h/e)	12.28 ± 4.71	−26.37 ± 1.89
46		L (h:ea/e)	61.84 ± 9.74	48.62 ± 1.18
47	*Schefflera morototoni* (Aubl.) Maguire, Steyerm. & Frodin	S (e)	42.73 ± 1.59	−23.89 ± 3.53
48		L (e)	9.05 ± 7.07	63.74 ± 8.13
49	*Solanum paludosum* Moric.	S (e)	17.69 ± 5.22	63.81 ± 2.08
50	*Xylopia frutescens* Aubl.	S (e)	19.36 ± 3.74	72.39 ± 2.90
51		L (m/e)	29.88 ± 2.90	18.19 ± 6.52
52		L (ea/e)	19.46 ± 7.46	51.24 ± 5.86
53		L (c/e)	71.00 ± 3.06	62.71 ± 4.30
54		L (h/e)	23.49 ± 3.57	69.41 ± 0.88
55	*Zanthoxylum rhoifolium* Lam.	L (e)	15.53 ± 5.03	14.00 ± 4.00
	Doxorubicin^e^		85.20 ± 0.78	86.34 ± 2.27

^a^Parts of the plant: *L* leaves, *S* stem, *F* Fruits, *FL* flowers

^b^Extractions: *h* hexane, *m* methanol, *e* ethanol, *m/e* methanol/water partition from the ethanolic extract, *ea/e* ethyl acetate partition from the ethanolic extract, *h/e* hexane partition from the ethanolic extract, *c/e* chloroform partition from the ethanolic extract, *h:ea/e*, hexane:ethyl acetate (1:1) partition from the ethanolic extract

^c^Cell lines: HepG2 (human hepatocellular carcinoma) and HL60 (human promyelocytic leukemia)

^d^GI % values are presented as the mean ± S.E.M. of two independents experiment performed in triplicate measured by the alamar blue assay after 72 h incubation. All extracts were tested at a concentration of 50 μg/mL. The negative control received the vehicle used to dilute the tested samples (0.5 % DMSO)

^e^Doxorubicin was used as the positive control

**Table 3:** IC_50_ values of plant extracts/fractions and isolated compounds against tumor cell lines

No.	Plant species	Parts of plant^a^ (Extractions^b^)	Cells^c^ (IC_50_ in μg/mL)^d^				
			B16-F10	HepG2	HL60	K562	PBMC
04	*Byrsonima sericea* DC.	L (m/e)	13.46	27.95	32.56	>50	33.81
			10.47–17.30	20.79–37.60	25.44–41.66		26.45–43.23
05		L (ea/e)	17.44	41.53	28.21	>50	>50
			13.95–21.81	35.31–48.85	21.18–37.57		
07		L (h/e)	16.89	26.19	16.14	28.32	17.58
			14.63–19.49	23.14–29.64	13.85–18.82	16.96–47.29	10.01–30.86
11	*Duranta repens* Linn.	L (c/e)	29.00	33.78	22.41	21.12	>50
			24.51–34.30	29.44–38.75	14.10–35.62	17.77–25.11	
56	Quercetin	-	12.12	12.15	8.81	14.93	14.39
			9.92–14.81	9.82–15.05	7.28–10.65	13.90–16.04	11.18–18.52
19	*Himatanthus bracteatus* (A.DC.) Woodson	L (c/e)	22.43	24.75	23.06	29.85	12.53
			20.80–24.18	21.70–28.22	20.62–25.79	26.87–33.15	8.28–18.96
20	*Ipomoea purga* (Wender.) Hayne	S (e)	7.39	8.52	9.39	8.53	6.99
			6.03–9.07	7.51–9.65	7.81–11.30	7.61–9.56	4.43–11.02
21		L (e)	16.30	29.63	32.64	28.95	30.71
			11.80–22.51	25.59–34.31	28.15–37.85	24.39–34.36	24.04–39.24
28	*Ixora coccinea* Linn.	F (c/e)	40.41	38.25	36.92	>50	>50
			36.60–44.63	30.46–48.04	33.52–40.67		
57	Mixture of α- and β-amyrin	-	23.21	24.09	>25	>25	Nd
			18.51–29.11	18.71–31.01			
29	*Mabea piriri* Aubl.	S (e)	17.76	32.98	22.72	21.89	>50
			13.42–23.51	27.61–39.40	13.26–38.91	15.52–30.87	
37	*Ocotea longifolia* Kunth (*Oreodaphne opifera* Mart. Nees)	L (e)	37.07	37.34	31.00	43.34	21.06
			32.10–42.81	31.34–44.49	21.04–45.69	39.47–47.58	17.48–25.37
43	*Psychotria capitata* Ruiz & Pav.	L (ea/e)	15.58	18.32	18.91	31.38	12.73
			11.29–21.51	14.91–22.50	13.93–25.67	22.78–43.22	7.43–21.81
44		L (c/e)	27.06	37.06	36.06	16.45	29.14
			19.26–38.02	31.54–43.53	28.65–45.40	13.16–20.56	22.46–37.82
	Doxorubicin^e^		0.08	0.03	0.02	0.06	0.14
			0.06–0.11	0.02–0.03	0.02–0.02	0.04–0.09	0.02–1.08

*Nd* Not determined

^a^Parts of the plant: *L* leaves, S stem, and *F* Fruits

^b^Extractions: *h* hexane, *m* methanol, *e* ethanol, *m/e* methanol/H_2_O partition from the ethanolic extract, *ea/e* ethyl acetate partition from the ethanolic extract, *h/e* hexane partition from the ethanolic extract, *c/e* chloroform partition from the ethanolic extract, *h:ea/e* hexane:ethyl acetate (1:1) partition from the ethanolic extract

^c^Tumor cells: B16-F10 (mouse melanoma), HepG2 (human hepatocellular carcinoma), HL-60 (human promyelocytic leukemia) and K562 (human chronic myelocytic leukemia). Non-tumor cells: PBMC (human peripheral blood mononuclear cells activated with concanavalin A – human lymphoblast*)*

^d^Data are presented as IC_50_ values in μg/mL and their 95 % confidence interval obtained by nonlinear regression from three independent experiments performed in duplicate, measured using alamar blue assay after 72 h incubation. The negative control received the vehicle used to dilute the tested samples (0.5 % DMSO)

^e^Doxorubicin was used as the positive control

In the preclinical cytotoxic drug screening program used in this work, which is based in the United States National Cancer Institute program, only those extracts that present IC_50_ values below 30 μg/mL and pure compounds with IC_50_ values below 4 μg/mL in tumor cell line assays are considered to be promising for anticancer drug development [9–11]. Therefore, the *B. sericea, D. repens, H. bracteatus, I. purga, I. coccinea, M. piriri, O. longifolia* and *P. capitata* extracts demonstrated promising cytotoxic activity. Doxorubicin, a clinical useful chemotherapy drug, was used as the positive control and presented IC_50_ values ranged from 0.02 to 0.08 μg/mL for HL-60 and B16-F10, respectively.

The pharmacology activity of *B. sericea* has been previously reported; the ethanolic extract from its leaves has gastroprotective properties that are mediated by nitric oxide and the K^+^ ATP channel. Phytochemical studies have revealed the presence of flavonoids (i.e., rutin, isoquercitrin, kaempferol 3-*O*-rutinoside and quercetin). However, its cytotoxic activity has not been previously reported [12]. Previous studies of *D. repens* have reported the presence of triterpenes, flavonoids, steroids, *C*-alkylated flavonoids and acetosides, as well as some alkaloids. Interestingly, durantanin IV and V and *E*/*Z* acteoside isolated from the leaves of *D. repens* have demonstrated significant cytotoxic activity against a HepG2 cell line [13]. Jalapinoside, a macrocyclic bisdesmoside resin glycoside, was recently isolated from the roots of *I. purga*, and its cytotoxic activity was evaluated. Jalapinoside was not cytotoxic (IC_50_ > 10 μg/mL), but reversal of multidrug resistance was observed using vinblastine-resistant human breast carcinoma cells [14]. In this study, we isolated the flavonol quercetin from the flowers of *D. repens,* which demonstrated a cytotoxic effect (with IC_50_ values ranging from 8.81 to 14.93 μg/mL on tumor cell lines HL-60 and K562, respectively). Quercetin has been previously reported as a cytotoxic agent because of its ability to induce apoptosis [15, 16].

The antitumor activity of flowers of *I. coccinea* was previously studied in murine models. Inhibited tumor growth and increased life spans were observed in Dalton’s lymphoma and Ehrlich ascites carcinoma-bearing mice [17]. Moreover, ixorapeptide I, a peptide derived from *I. coccinea*, displayed cytotoxicity against the Hep3B liver tumor cell line, with an IC_50_ value of 3.36 μg/mL [18]. Interestedly, although the use of *I. coccinea* is currently only ornamental in Brazil, its flowers are an ingredient in an Ayurvedic cancer formulation in India [19, 20]. The flowers of *I. coccinea* are used to treat cancer, leucorrhoea, dysentery, dysmenorrhea, hemoptysis and hypertension; its leaves are used to pacify vitiated pitta, skin diseases, colic, flatulence, diarrhea, indigestion, ulcers and wounds, and they are also used as an antiseptic [20, 21]. The roots of *I. coccinea* are used as an astringent and antiseptic against scabies and other skin diseases [20]. In this study, the fractionation of the extract of *I. coccinea* flowers resulted in the mixture of α- and β-amyrin, which demonstrated weak cytotoxic activity toward tumor cell lines. In fact, no potent cytotoxic activity has been reported for these compounds.

Prieto et al. [22] have reported that essential oil from the leaves of *O. longifolia*, of which α-terpinolene and α-phellandrene are the primary ingredients, demonstrates antifungal activity against *Sitophilus zeamais*. However, the cytotoxic activity of the *O. longifolia* plant has not been previously reported. No previous biological effects have been reported for the medicinal plants *H. bracteatus, M. piriri* and *P. capitata*.

The extracts from *C. impressinervia*, *H. tomentosa*, *M. minutiflora*, *M. charantia*, *O. glomerata*, *P. fruticosa*, *S. morototoni*, *S. paludosum*, *X. frutescens* and *Z. rhoifolium* showed no pronounced cytotoxic effects. Among these plants, essential oil from the leaves of *X. frutescens* has been previously assessed for its cytotoxic and antitumor effects; the oil demonstrated potent in vitro cytotoxic activity against tumor cell lines from different histotypes and in vivo antitumor activity in sarcoma 180 murine model [23]. Although essential oil from *Z. rhoifolium* leaves and *M. charantia* fruit extract have been previously assessed for cytotoxicity, they demonstrated no cytotoxic effects [24, 25]. The cytotoxic potential of *C. impressinervia*, *H. tomentosa, M. minutiflora, O. glomerata, P. fruticose, S. morototoni* and *S. paludosum* plants has never been investigated.

## Conclusion

In conclusion, we screened the cytotoxic potential of 55 extracts from 18 plants found in northeast Brazil, and extracts from *B. sericea, D. repens, H. bracteatus, I. purga, I. coccinea, M. piriri, O. longifolia* and *P. capitata* demonstrated potent cytotoxic effects. The fractionation of *D. repens* and *I. coccinea* extracts led to the isolation of quercetin and the mixture of α- and β-amyrin, respectively, and quercetin showed moderate cytotoxic activity. Fractionation of the other plants should be performed to identify their active constituents, and further investigations are required to determine their mechanisms of cytotoxicity and in vivo activities. This work reinforces the need to understand the therapeutic potentialities of Brazilian medicinal plants.

## Abbreviations

CC, column chromatography; ConA, concanavalin A; DMSO, dimethyl sulfoxide; IC_50_, 50 % inhibitory concentration; NMR, nuclear magnetic resonance; PBMC, peripheral blood mononuclear cells; RPMI-1640, roswell park memorial institute-1640; TLC, thin layer chromatography; TMS, tetramethylsilane.
